# Bio-Energy Retains Its Mitigation Potential Under Elevated CO_2_

**DOI:** 10.1371/journal.pone.0011648

**Published:** 2010-07-19

**Authors:** Marion Liberloo, Sebastiaan Luyssaert, Valentin Bellassen, Sylvestre Njakou Djomo, Martin Lukac, Carlo Calfapietra, Ivan A. Janssens, Marcel R. Hoosbeek, Nicolas Viovy, Galina Churkina, Giuseppe Scarascia-Mugnozza, Reinhart Ceulemans

**Affiliations:** 1 Department of Biology, University of Antwerp (UA), Wilrijk, Belgium; 2 Laboratoire des sciences du climat et de l'environnement (LSCE), Unité mixte (UM) de Commissariat à l'énergie atomique et aux énergies alternatives (CEA), Centre national de la recherche scientifique (CNRS) and Université de Versailles Saint Quentin Yvelines (UVSQ), Gif sur Yvette, France; 3 Natural Environment Research Council (NERC) Centre for Population Biology, Imperial College London, Ascot, United Kingdom; 4 Institute of Agro-Environmental and Forest Biology (IBAF), National Research Council (CNR), Monterotondo Scalo, Italy; 5 Department of Environmental Sciences, Earth System Science – Climate Change, Wageningen University, Wageningen, The Netherlands; 6 Leibniz-Centre for Agricultural Landscape Research (ZALF), Muencheberg, Germany; University of Zurich, Switzerland

## Abstract

**Background:**

If biofuels are to be a viable substitute for fossil fuels, it is essential that they retain their potential to mitigate climate change under future atmospheric conditions. Elevated atmospheric CO_2_ concentration [CO_2_] stimulates plant biomass production; however, the beneficial effects of increased production may be offset by higher energy costs in crop management.

**Methodology/Main Findings:**

We maintained full size poplar short rotation coppice (SRC) systems under both current ambient and future elevated [CO_2_] (550 ppm) and estimated their net energy and greenhouse gas balance. We show that a poplar SRC system is energy efficient and produces more energy than required for coppice management. Even more, elevated [CO_2_] will increase the net energy production and greenhouse gas balance of a SRC system with 18%. Managing the trees in shorter rotation cycles (i.e., 2 year cycles instead of 3 year cycles) will further enhance the benefits from elevated [CO_2_] on both the net energy and greenhouse gas balance.

**Conclusions/Significance:**

Adapting coppice management to the future atmospheric [CO_2_] is necessary to fully benefit from the climate mitigation potential of bio-energy systems. Further, a future increase in potential biomass production due to elevated [CO_2_] outweighs the increased production costs resulting in a northward extension of the area where SRC is greenhouse gas neutral. Currently, the main part of the European terrestrial carbon sink is found in forest biomass and attributed to harvesting less than the annual growth in wood. Because SRC is intensively managed, with a higher turnover in wood production than conventional forest, northward expansion of SRC is likely to erode the European terrestrial carbon sink.

## Introduction

Continuously rising atmospheric CO_2_ concentration ([CO_2_]) and the depletion of fossil fuel stocks has created a demand for secure supplies of carbon-neutral substitute fuels. A variety of biofuels based on food crops, such as bio-ethanol from grain or corn, and bio-diesel from soya were considered as viable alternatives to fossil fuels, but recent studies have identified adverse environmental effects that compromise their climate change mitigation potential [1], [2], [3].

However, a new generation of biofuels produced from ligno-cellulosic compounds of non-food crops such as grasses and woody crops are now candidates for wide scale planting. These biofuels are thought to have a higher mitigation potential and more beneficial socioeconomic effects compared to biofuels based on food crops. Their production does not necessarily compete with food crops for the most fertile soils and their management is usually less intensive than that applied to food crop based biofuel [3], [4], [5].

If bioenergy is to supply a substantial share of the future energy demand [6], its potential to mitigate climate change should be evaluated not only under current ambient but also under future elevated atmospheric [CO_2_]. Elevated [CO_2_] is commonly observed to stimulate biomass growth [7], especially when there is an ample supply of water and nutrients [8], [9]. However, gains in energy yield may be offset by greater handling costs and the need for more intensive crop management to maintain productivity.

For six years, we fumigated a poplar short rotation coppice (SRC) plantation with elevated [CO_2_] (550 ppm) using Free Air CO_2_ Enrichment (FACE) technology (the POP/EUROFACE experiment. Here we present, for the first time to the best of our knowledge, the net energy balance (NEB; the difference between the energy output and consumption of the SRC) and greenhouse gas balance (GHGB; net amount of CO_2_ and other greenhouse gases, expressed in CO_2_ equivalents, removed from or released into the atmosphere during the life cycle of the SRC) of a full-scale poplar plantation grown under current-ambient and future-elevated [CO_2_].

## Results

**Figure 1:**
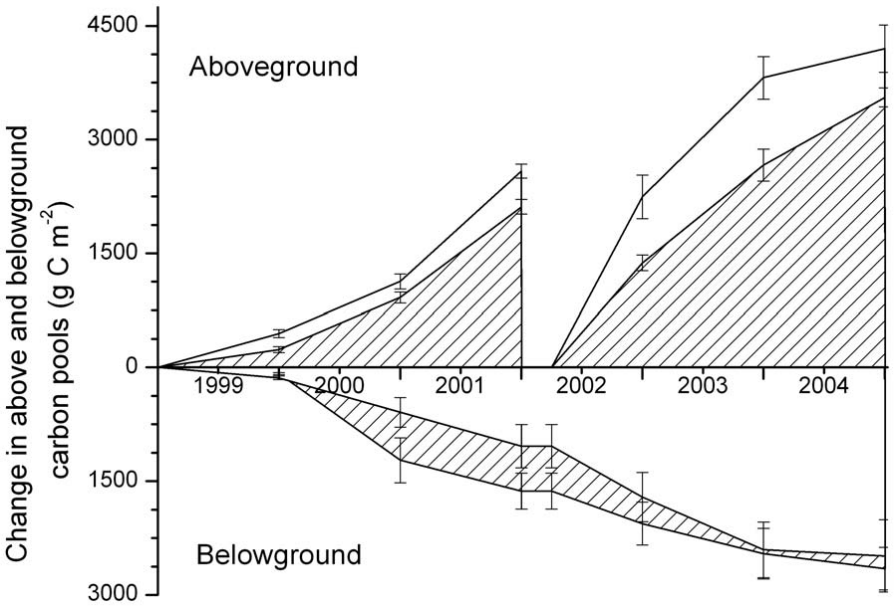
Change in above- and belowground ecosystem carbon (C) storage and its standard error (g C m^−2^) in a poplar short rotation coppice system (SRC) growing under ambient (checked area) and elevated (white area) [CO_2_]. Carbon storage aboveground consisted of the carbon in stems and branches that were harvested every three years for the production of bio-energy. Belowground carbon storage, shown below the x-axis to stress its belowground character but indicating an increase, was the total sum of the carbon contained in the fine and coarse roots, stumps, litter and the soil. Average yield in ambient and elevated [CO_2_] was 44 and 53 ton DM ha^−1^ respectively after the first rotation and 74 and 87 ton DM ha^−1^ after the second rotation. Data adapted from [12], [24], [25], [26], [27], [35].

The first harvest yielded on average 22% more harvestable biomass in the future elevated [CO_2_] treatment than in the current ambient [CO_2_] (F = 6.72, P<0.05) (Figure 1). Coppicing the trees increased aboveground production and by the end of the second rotation harvestable biomass yield was 18% higher under elevated [CO_2_] (F = 4.58, P<0.05). During the first rotation, elevated [CO_2_] enhanced the biomass of stumps, coarse roots and fine roots (respectively: F = 44.5, P<0.01; F = 13.1, P<0.01; F = 22.7, P<0.01). The [CO_2_]-induced stimulation of fine root biomass disappeared in the second rotation (F = 0.99, P>0.1), but stumps and coarse root systems remained larger. Despite [CO_2_]-induced stimulation of soil carbon inputs [10] soil carbon sequestration was suppressed during the first rotation (F = 9.91, P<0.01). This was likely due to a priming effect, where the additional labile carbon increased the decomposition of older carbon [10], [11], [12]. Following the first harvest, priming ceased and in subsequent years soil carbon built up more rapidly under elevated [CO_2_] to reach, by the end of the observational period, a soil carbon content similar to the content under ambient [CO_2_] (Figure 1).

Based on these observations we simulated the life cycle of a poplar SRC from four different scenarios. Each scenario consisted of six rotations and in all 4 scenarios trees were harvested after three years during the first rotation cycle. We varied the length of the following 5 rotation cycles (i.e. either three or two years per rotation), such that the total life cycle was 18 or 13 years respectively. Both management strategies were applied under current ambient and future elevated [CO_2_] thus resulting in a total of four different scenarios (Table 1).

**Table 1:** Greenhouse gas (GHG) reduction (positive values) or release (negative value) of a poplar SRC (ton CO_2_-equivalent ha^−1^) (± SD) under current and elevated [CO_2_], managed for six rotations of two or three years.

	18 yrs: 3 year rotation		13 yrs: 2 year rotation	
	Current [CO_2_]	Elevated [CO_2_]	Current [CO_2_]	Elevated [CO_2_]
N_2_O emissionΔ	−14 (±1)	−17 (±1)	−11 (±1)	−16 (±1)
CH_4_ mitigationδ	0.050 (±0.01)	0.050 (±0.01)	0.050 (±0.01)	0.050 (±0.01)
CO_2_ release from biomass production‡	−62 (±3)	−77 (±4)	−49 (±2)	−71(±4)
Avoided CO_2_ by displacing fossil fuels^ε^	701 (±29)	830 (±34)	543 (±22)	763 (±32)
Net GHG reduction from bio-energy production	625 (±26)	737 (±31)	484 (±20)	677 (±28)
Net yearly GHG reduction from bio-energy production	35 (±1)	41 (±2)	37 (±2)	52 (±2)

Δ N_2_O emission from fertilization is calculated as a loss of 4% (30) from the amount of fertilizer added (see Table S5).

δ Values for CH_4_ mitigation were taken from [37].

‡ Cumulative sum of all fixed and variable costs during the course of the full life cycle (Table S1, S3, S4, and S5).

The observed evolution of soil carbon is not an inherent property of the SRC system and was therefore omitted from the calculations. Hence, our calculations underestimate the beneficial effects of SRC when planted on former agricultural lands. We assumed a combined heat and power biomass plant displaces a combined heat and power coal plant with an emission of 103 g CO_2_ MJ^−1^ for the combined heat and electricity and 121 g CO_2_ MJ^−1^ for just the electricity production [36]. Since coal is among the most GHG emitting fuels, avoided emissions approximate the maximum possible avoided emissions. A combined heat and power gas plant emits 59 CO_2_ MJ^−1^ for its combined heat and electricity and 70 g CO_2_ MJ^−1^ for its electricity production [36]. The GHGB and mitigation potential for gas instead of coal substitution is given in Table S2.

We estimated the gross energy production (i.e. biomass yield multiplied with the energy content of the wood) for a SRC system growing for six three-year rotations under ambient [CO_2_] at 444±18 GJ ha^−1^ yr^−1^ (Calculations are detailed in the supporting information Methods and material S1 and Tables S1, S2, S3, S4, and S5). CO_2_ enrichment stimulated biomass production and thus gross energy yield by 18%, up to 526±22 GJ ha^−1^ yr^−1^. The overhead in energy needed to manage, harvest, transport and finally convert woody biomass into bio-energy increased from 32±1 GJ ha^−1^ yr^−1^ under current conditions to 39±2 GJ ha^−1^ yr^−1^ under elevated [CO_2_]. Consequently, the energy efficiency of a poplar SRC, expressed as the gross energy production over its consumption, was estimated at 14±1 and was not affected by [CO_2_] up to 550 ppm. Hence, for every unit of energy needed to manage the SRC, 14 units of energy are produced.

Biomass conversion into heat and electricity in a combined heat and power biomass firing plant, would generate a net amount of energy of 346±14 GJ ha^−1^ yr^−1^ under current ambient and 409±17 GJ ha^−1^ yr^−1^ under future elevated [CO_2_]. Elevated [CO_2_] thus enhances the NEB of combined heat and power proportionally to the increase in biomass production (i.e. 18%, P-value of permutation test <0.01; Figure S1a). The net energy efficiency decreases to 11±1 compared to its gross energy efficiency of 14±1, due to energy losses in the combined heat and power plant. Advancing harvest by managing the trees in shorter rotations of two instead of three years increases the system NEB by 7 and 27% in respectively current ambient and future elevated [CO_2_] (P<0.01, Figure S1). Moreover, the current NEB of a poplar SRC could increase by 50% if plantations grown under future elevated [CO_2_] are managed in two year rotation cycles (P<0.01). The statistical significance of the positive [CO_2_] effect on NEB is sustained for uncertainties in biomass production up to 15% and was insensitive to uncertainties in the conversion factors (Figure S2).

Given a per capita energy consumption of 158 GJ in Europe [13] 1.2 and 0.9 hectares of SRC would be required per capita to satisfy this need under respectively current ambient and future elevated atmospheric [CO_2_]. However, the productivity and thus NEB observed at our site more likely represents the maximum rather than average productivity that can be achieved with SRC in Europe. Hence, the areas reported above are minimal requirements.

Under current [CO_2_], the GHGB of a fertilized and irrigated poplar SRC system was positive and thus using the biomass from our SRC system in a combined heat and power plant removed a net amount (or avoided CO_2_ emission) of 35±1 ton CO_2_-equivalent ha^−1^ yr^−1^ from the atmosphere when compared to coal (Table 1, or Table S2 for a comparison to natural gas). Growing poplars under elevated [CO_2_] increased the positive GHG balance and bioenergy from SRC avoided annually 41±1 ton CO_2_-equivalent ha^−1^ from being emitted into the atmosphere (P-value of permutation test <0.01). In the future, the GHGB could be significantly enhanced (P-value of permutation test <0.01) up to 52±1 ton CO_2_-equivalent ha^−1^ by reducing the rotations length to two years under elevated [CO_2_]. These results hold for all realistic uncertainty settings (Figure S3).

A SRC is greenhouse gas neutral if it produces exactly the amount of biomass that is required to have the avoided emissions compensate for the total emissions from crop management and bio-energy production. A SRC that produces less biomass has a negative GHGB and thus emits GHG to the atmosphere. We estimated the biomass production required to render a GHG neutral SRC under the four different life cycles considered in this study. The GHGB was calculated for a SRC with an assumed biomass production of 1 ton DM ha^−1^ yr^−1^ and was found to be negative. Subsequently the assumed biomass production was increased by 1 ton DM ha^−1^ yr^−1^ until the GHGB became positive. A minimum production of on average 3.2±0.1 ton DM ha^−1^ yr^−1^ was found needed to obtain a neutral GHGB under elevated [CO_2_] coppiced in two-year rotations, whereas on average 2.0±0.1 ton DM ha^−1^ yr^−1^ results in a neutral GHGB under current ambient [CO_2_] in three-year rotations.

**Figure 2:**
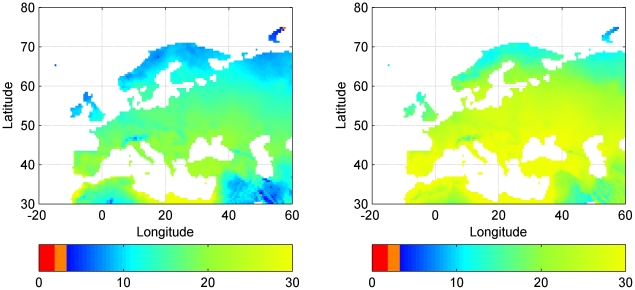
Biomass production (ton DM ha^−1^ yr^−1^) of fertilized and irrigated poplar SRC in Europe. Whether the predicted biomass production can be realized at a given location will depend on the availability of nutrients and water. Red and orange indicate production levels for which an SRC emits more GHG than it absorbs (a) Areal extent of GHG-neutral SRC system under 1991–2000 [CO_2_] and climate conditions. Under current conditions the minimal biomass production to obtain a GHG-neutral bio-energy system was estimated at 2.0±0.1 ton DM ha^−1^ yr^−1^ (production indicated as red). (b) Areal extent of GHG-neutral SRC system under future [CO_2_] and climate conditions (IPCC scenario A1B in 2059–2068). Under these conditions the minimal biomass production to obtain a GHG-neutral bio-energy system was estimated at 3.2±0.1 ton DM ha^−1^ yr^−1^ (production indicated as red plus orange).

Subsequently, we used the ecosystem model ORCHIDEE-FM [14] to simulate current and future biomass production of fertilized and irrigated poplar SRC systems across Europe. If fertilized and irrigated the GHGB of SRC is always positive which does not necessarily imply that the SRC is also economically feasible. Despite the fact that future atmospheric conditions require higher biomass production per unit land area to become GHG-neutral, future conditions are expected to result in an increased biomass production. The increase in biomass production compensates the higher biomass production requirements for an SRC to become GHG-neutral. Higher future biomass production is expected to result in a northward extension of the area where SRC may mitigate climate change through reduced emissions from fossil fuel burning (Figure 2).

The results presented above exclude the soil carbon dynamics. Soil carbon dynamics were omitted from the GHGB estimates of the SRC because whether the soil is a sink or source of carbon depends on site history and is therefore not an inherent characteristic of the SRC system. We used model simulations from ORCHIDEE-FM, BIOME-BGC and when available site observations from the POP/EUROFACE experiment to quantify changes in soil carbon content (Table 2). Changes in soil carbon are largest the first decades following a land-use change. Accounting for the changes in soil carbon in the GHGB of our field site resulted in an increase in the GHGB of 3 ton CO_2_-equivalents ha^−1^ y^−1^ for the first 18 years following the conversion of a maize cropland to a SRC. Over a century the increase in the GHGB would reduce by 0.7 to 1.0 ton CO_2_-equivalents ha^−1^ y^−1^ under respectively future and current atmospheric [CO_2_]. Over a millennium, the time required to reach equilibrium in the soil carbon pools, the increase in the GHGB would be less than 0.3 ton CO_2_-equivalents ha^−1^ y^−1^ (Table 2).

**Table 2:** Observed (POP/EUROFACE) and modeled (ORCHIDEE-FM and BIOME-BGC) changes in soil carbon (ton CO_2_ ha^−1^) under current ambient and future elevated [CO2].

Time since conversion (Years)	Source	Forest		Grassland		Cropland (Maize)	
		Current [CO_2_]	Elevated [CO_2_]	Current [CO_2_]	Elevated [CO_2_]	Current [CO_2_]	Elevated [CO_2_]
6	POP/EUROFACE	n.a.	n.a.	n.a.	n.a.	+18	+18
18	ORCHIDEE-FM	−1	−28	−84	−106	+35	+25
18	BIOME-BGC	−6	−6	−12	−12	n.a.	n.a.
100	ORCHIDEE-FM	−137	−178	−152	−187	+101	+71
100	BIOME-BGC	−20	−17	−30	−26	n.a.	n.a.
1000	ORCHIDEE-FM	−281	−347	−229	−294	+243	+178

Changes are reported for 6, 18, 100 and 1000 years since land-use change from forest, grassland and a maize cropland to SRC with poplar.

## Discussion

By the end of the second rotation harvestable biomass yield was 18% higher and also growth of stumps and coarse roots had increased under elevated [CO_2_]. About 40% of the newly sequestered carbon was stored belowground. However, at the end of a full life cycle of a poplar SRC, stumps and coarse roots must be removed to allow other crops to be planted or to start the next SRC cycle with new plants. Any carbon accumulation in stumps and coarse roots is therefore ephemeral in this type of system; soil carbon is the only belowground carbon pool of which a part may have a residence time longer than the SRC life cycle. Whether the soil will act as a carbon sink depends on the pre-SRC type of land-use. If forests, peatlands or grasslands are converted into SRC, conversion causes a carbon debt [1] partly offsetting the greenhouse gas benefit of SRC (Table 2). Consequently, direct or indirect clearing to release land for SRC should be avoided. On the other hand, converting degraded or abandoned agricultural lands to SRC is likely to result in carbon sequestration [15], as observed in our experiment (Figure 1 and Table 2).

The trends in soil carbon accumulation we observed depend on the former land-use of the site and are therefore not an inherent property of the SRC system. Over an 18 year time frame, the presented GHGB may decrease (forests, peatlands or grasslands) or increase (i.e. for degraded or abandoned agricultural lands) by 5 to 15% when accounting for soil carbon dynamics. When accounted for over century long periods the effect on the GHGB balance drops below 5%. Moreover, while ORCHIDEE correctly simulates qualitative trends in soil carbon following land-use changes, its predictive power is limited in terms of absolute values and time dynamics. Consequently, observed and simulated soil carbon dynamics (Table 2) were omitted from further calculations of the GHGB. Our calculations thus underestimate the beneficial effects of SRC when it is planted on former agricultural lands.

Life cycle analysis was used to evaluate the viability of this poplar SRC as a future biofuel for combustion in a combined heat and power plant. Over the life cycle of the SRC the gross energy output increased proportional to the increase in biomass production (i.e. by 18%). At the same time not only the fuel overhead but also fertilization costs increased both in proportion to the increase in biomass under elevated [CO_2_]. Despite the generally observed reduction in leaf-level stomatal conductance under elevated [CO_2_] [16], site-level transpiration of the SRC was observed to increase by up to 23% owing to a larger leaf area index, the integrated plant response to light and vapor pressure deficit and the lengthening of the growing season under elevated compared to current [CO_2_] [17]. Increased water demand was met by increased irrigation, and thus irrigation costs increased faster than the increase in biomass under elevated [CO_2_]. Based on stem-flow observations for the SRC plantation [17] and our estimates for NEB (see below), the water footprint of poplar SRC reaches 42 m^3^ GJ^−1^ under current and 44 m^3^ GJ^−1^ under elevated [CO_2_]. Despite irrigation, SRC with poplar thus ranks among the most efficient bioenergy crops in terms of water use [18].

The NEB of the poplar SRC under current [CO_2_] (i.e. 346±14 GJ ha^−1^ yr^−1^) is much higher than corn-based ethanol (18.9 GJ ha^−1^ yr^−1^ [19], soy-based bio-diesel 14.4 GJ ha^−1^ yr^−1^ [19]), and low input systems such as high diversity grasslands 17.8 to 28.4 GJ ha^−1^ yr^−1^ [3] and switch grass plantations 60 GJ ha^−1^ yr^−1^ [5]). The positive NEB of the poplar SRC resulted from a yield 150 to 1000% larger than yields from other biofuel crops and the ability to use all aboveground biomass for energy production.

Elevated [CO_2_] enhanced the NEB and an irrigated and fertilized poplar SRC is thus energy positive, yielding 14±1 times more energy than is needed for the intensive production process. The energy efficiency of the SRC dropped to 11±1 when the energy losses in a combined heat and power plant were accounted for. The observed interaction between [CO_2_] and rotation length indicates that elevated [CO_2_] may enhance NEB by 50% if the plantation were to be managed in shorter rotation cycles. Elevated [CO_2_] accelerated canopy development, causing the onset of light competition to advance from the third to the second year of the post-harvest cycle. Harvesting the biomass just before light-induced mortality maximizes the net energy balance. Optimizing the benefits from increasing [CO_2_] may therefore require an adapted SRC management strategy.

When estimating the GHGB (expressed in metric ton CO_2_-equivalent ha^−1^ yr^−1^), we included the CO_2_ emissions from biomass production, methane (CH_4_) oxidation and nitrous oxide (N_2_O) emissions together with the fossil fuel emissions avoided by substituting coal with poplar biomass to fire a combined heat and power plant (Table 1). Growing poplars under elevated [CO_2_] resulted in a more positive GHG balance compared to the GHGB under current ambient conditions. Substitution of fossil fuels by the production and use of bio-energy from a poplar SRC effectively avoids greenhouse gas emissions to the atmosphere.

The mitigation potential of a biofuel production system can be quantified by the ratio of its GHGB and NEB, and is thus a measure of the amount of avoided greenhouse gas emissions for every net MJ of energy produced. With 84±1 g CO_2_-equivalent avoided emissions per produced MJ, the mitigation potential of a poplar SRC currently takes an intermediate place in the ranking (Table S6). In contrast, the production of liquid biofuels such as corn ethanol, soy biodiesel, and switch grass ethanol are net carbon sources, but still contribute less to global warming than their fossil fuel counterparts. Even when the SRC management strategy is aimed at maximizing its NEB i.e. through fertilization and irrigation, its potential to mitigate climate change remains favorable. Although both the net energy and GHG balance of a poplar SRC under future atmospheric conditions could benefit from shorter rotations, its mitigation potential does not change. Consequently, by altering management practice, a poplar SRC can be adapted to future atmospheric conditions without jeopardizing its mitigation potential.

Future atmospheric conditions require higher biomass production (3.2±0.1 vs. 2.0±0.1 ton DM ha^−1^ yr^−1^) per unit land area to become GHG-neutral. The larger GHG cost of SRC under elevated CO_2_ is explained by the more frequent and thus more costly harvesting and irrigation. The future increase in potential biomass production due to elevated [CO_2_] outweighs the increased production costs resulting in a northward extension of the area where SRC is GHG-neutral (Figure 2). Currently, the main part of the European terrestrial carbon sink takes the form of standing forest biomass [20] and this sink has been attributed to harvesting less than the annual growth in wood [21]. Because SRC is intensively managed, with a higher turnover in wood production than conventional forest, northward expansion of SRC is likely to erode the European terrestrial carbon sink.

Although fertilized and irrigated poplar SRC shows to be a viable substitute for fossil fuels under both current ambient and future elevated atmospheric [CO2], its application can lead to, if used in certain regions, unintended environmental impacts such as withdrawing land, water and fertilizer from food production [15] or eroding the European terrestrial carbon sink. Therefore, only a diversification within the different forms of sustainable source of energy may guarantee the replacement of our ending fossil fuels.

## Materials and Methods

For six years, we fumigated a poplar short rotation coppice (SRC) plantation with elevated [CO_2_] (550 ppm) using Free Air CO_2_ Enrichment (FACE) technology (the POP/EUROFACE experiment; http://www.unitus.it/dipartimenti/disafri/progetti/euroface/). Poplar is a fast growing species commonly grown where the water table is close to the surface. After cutting, poplar re-grows from the stump, making it amenable to coppicing and later mechanical harvesting. The experimental facility was located in central Italy (latitude 42°37′40″N, longitude 11°08′87″E, altitude 150 m). Dense stands (10,000 trees ha^−1^) of three poplar species (*Populus alba*, *P. nigra* and *P. x euramericana*) were planted on 9 ha of fertile former agricultural land where the initial soil nitrogen content reached 7.7 to 10.4 µg N g^−1^ soil [22]. The experimental plantation was irrigated throughout each summer. After three years, aboveground biomass was harvested establishing a multi-stem coppice for the following rotation.

Annual above- and belowground biomass production were estimated by selective harvests [23], [24], [25] and root coring [26], respectively. At the end of each rotation, aboveground biomass was harvested [22], [23], [24] and belowground biomass was estimated by site-specific allometric relationships parameterized by data from excavated roots and stumps. Carbon storage in soils was analyzed annually (except for 2002) from soil cores [12], [27].

Life cycle analysis was used to evaluate the viability of this poplar SRC as a future biofuel. We estimated the NEB and GHGB of a full life cycle of a poplar SRC, consisting of six rotations, each of either two or three years duration. Cutting frequency determined the maximum length of the poplar life cycle, as the exhaustion of stump carbohydrate reserves after more than six cutting cycles jeopardizes re-growth [28]. Biomass yield was based on the observed productivity from the six year POP/EUROFACE experiment averaged across three poplar species. We assumed that the productivity in rotations three to six would be similar to the ones observed during the second rotation [29]. We quantified the inputs and outputs of energy and GHGs (Table S1) from published energy costs and conversion factors (Tables S3, S4, and S5). To account for the numerous assumptions made, we propagated uncertainties through the calculations of NEB and GHGB by running 10,000 random realizations based on Monte Carlo principles.

We used the ecosystem model ORCHIDEE-FM [14] extended with a new forest management module to simulate the biomass production at our site (Figure S4) and of fertilized and irrigated poplar SRC systems across Europe. The biomass production was simulated for: (i) current [CO_2_] and average climate conditions between 2000–2009 and (ii) future [CO_2_] and climate conditions in 2059–2068 according to IPCC scenario A1B [30].

BIOME-BGC is a process model describing the carbon, nitrogen and water cycles [31] of land ecosystems. It has been corroborated for a number of hydrological and carbon cycle components as well as for forest management [32], [33], [34]. Both ORCHIDEE-FM and BIOME-BGC were used to simulate changes in soil carbon associated with conversion of forests and grasslands to poplar plantation. First, we performed spinup simulation of the model for deciduous broad leaf forest, C3 grassland and C4 cropland (only with ORCHIDEE-FM). Then transient simulations were performed for a plantation of deciduous broadleaf forest.

## Supporting Information

Methods and Material S1 (0.08 MB DOC)

Table S1 Components of the full life cycle analysis of poplar SRC for biomass production under current ambient and future elevated [CO_2_] and subsequent combustion in a combined heat and power plant. Avoided CO_2_ emissions, GHGB and mitigation potential were based on the assumption that coal was substituted by biomass in a combined heat and power plant. Excluding soil carbon dynamics (see Table 2).
(0.12 MB DOC)

Table S2 Components of the life cycle analysis of poplar SRC for biomass production under current ambient and future elevated [CO_2_] and subsequent combustion in a combined heat and power plant. Avoided CO_2_ emissions, GHGB and mitigation potential were based on the assumption that gas was substituted by biomass in the combined heat and power plant. Excluding soil carbon dynamics (see Table 2).
(0.05 MB DOC)

Table S3 Estimated fixed energy costs (GJ ha^-1^) for planting, growing and maintaining a poplar SRC.
(0.05 MB DOC)

Table S4 Machinery utilized for field operations in a poplar SRC. Data from [24] and [23].
(0.03 MB DOC)

Table S5 Estimated variable energy costs.
(0.04 MB DOC)

Table S6 Greenhouse gas mitigation potential (i.e. GHG sequestration (positive) or release (negative) per net energy gain (g CO_2_ equivalent MJ^-1^) for different bio-fuels and their respective fossil fuel counterparts. The mitigation potential of a biofuel production system can be quantified by the ratio of its GHGB and NEB, and is thus a measure of the amount of avoided greenhouse gas emissions (CO_2_-equivalent) for every net MJ of energy produced.
(0.04 MB DOC)

Figure S1 Simulated life cycle of net energy gain (GJ) from a poplar SRC growing for 6 rotations under different coppice regimes in current and elevated [CO_2_]. a) Current and elevated [CO_2_] grown poplars managed in three year rotations b) current [CO_2_] grown poplars managed in three year rotation cycles, elevated [CO_2_] grown trees in two year rotation cycles, c) both current and elevated [CO_2_] grown trees are managed in two year rotation cycles. The grey area shows the 95% uncertainty interval.
(0.95 MB TIF)

Figure S2 Sensitivity analysis of NEB to uncertainties in biomass production and conversion factors (Tables S1, S3, S4, S5). (a) Blue pixels shows uncertainty settings for which NEB under elevated is significantly higher than NEB under current [CO_2_]. Green pixels show uncertainty settings for which no significant differences were found. (b) Similarly, blue pixels show uncertainty settings for which NEB for two year rotations is significantly higher than NEB for three year rotations. (c) Whether [CO_2_] and management have a significant effect on the NEB of an SRC depends on the uncertainty in biomass production. Uncertainties in biomass production below 15% will result in a significant effect, irrespective of the uncertainty in the conversion.
(3.87 MB TIF)

Figure S3 Sensitivity analysis of the GHGB to uncertainties in biomass production and conversion factors (Tables S1, S3, S4 and S5). In the combined heat and power plant coal is substituted by biomass. (a) Blue pixels show uncertainty settings for which poplar SRC under elevated [CO_2_] removes significantly more CO_2_-equivalents from the atmosphere than under current [CO_2_]. Green pixels shows uncertainty settings for which no significant differences were found. (b): Settings for which GHGB for two year rotations is significantly higher than GHGB for three year rotations (c) Blue pixels show uncertainty settings for which poplar SRC under elevated [CO2] and with two year rotations removes significantly more CO_2_-equivalent from the atmosphere than under current [CO_2_] with three year rotations.
(3.87 MB TIF)

Figure S4 Comparison of model output of ORCHIDEE-FM against observed aboveground biomass production. (a) Comparison for ambient [CO_2_] (b) Comparison for elevated [CO_2_]. Small decreases in biomass are due to modeled reserve mobilization to subsidize growth in the following spring. The larger decrease in biomass in the second year of the second rotation is due to the onset of competition, the current model version, accounts for this loss of biomass on the last day of the year. For both comparisons, the climate data driving simulations come from the 0.25° resolution REMO reanalysis, which covers Europe from 1861 to 2007.
(0.10 MB TIF)
