# Supplementary material for: Bio-Energy Retains Its Mitigation Potential Under Elevated CO2
Source: PLoS One. 2010 Jul 19;5(7):e11648. doi: 10.1371/journal.pone.0011648 (PMC2906505; doi:10.1371/journal.pone.0011648)
Supplement: Methods and Material S1 — (0.08 MB DOC) [file pone.0011648.s001.doc]

**Supporting information**

**Abbreviations:**

**[CO_2_]**: atmospheric CO_2_ concentration.

**DM**: dry matter.

**GHGB**: Greenhouse Gas Balance. The net amount of CO_2_ and other greenhouse gases (expressed in metric ton CO_2_-equivalent ha^-1^ yr^-1^), removed from or released into the atmosphere during the production and consumption of bio-energy from a poplar short rotation coppice.

**NEB**: Net Energy Balance. The NEB (expressed in joules (J)) is calculated as the difference between the gross energy content of the produced biomass and the total energy consumption for facilitating coppice management. The energy efficiency (dimensionless) of the SRC is calculated as the ratio of the gross energy content of the produced biomass and the total energy consumption for facilitating coppice management.

**SRC**: Short Rotation Coppice. In this manuscript the use of this abbreviation implies short rotation coppice with poplar.

**Experimental design:** We used the POP/EUROFACE results as an observational data set to estimate the net energy balance (NEB) and greenhouse gas balance (GHGB) of a poplar short rotation coppice system growing under current and future elevated CO_2_ concentrations [CO_2_].

The POP/EUROFACE experimental field was planted in 1999 with poplar cuttings of three different species (*Populus alba*, *P. nigra* and *P. x euramericana*) on former agricultural land at a density of 10,000 trees ha^-1^. Six experimental plots were identified as having uniform soil conditions, and in three of them a FACE design was installed, providing the trees with daytime elevated CO_2_ concentrations of 550 ppm. Half of each plot was fertilized with 212 kg ha^-1^ of N-P_2_O_5_-K_2_O: 20-6-6 during the first year and 290 kg N (ammonium nitrate: 34-0-0) ha^-1^ during the second and third years. Trees were irrigated throughout each growing season and were mechanically weeded during the first years after planting and coppicing to ensure tree survival. A detailed description of the design and performance of the FACE fumigation system can be found in [1].

Allometric relations [2,3,4] from a selective harvest were used to estimate yearly aboveground biomass production. After three years, aboveground woody biomass was harvested. At the beginning of the second rotation cycle, numerous shoots emerged from the coppiced stumps left after the first rotation cycle. Three years later, thus at the end of the second three-year growth cycle, another aboveground woody biomass harvest was carried out. Belowground productivity (i.e. production of fine and coarse root biomass, 0-40 cm depth) was monitored using ingrowth cores and sequential coring [5]. A sub-sample of stumps and roots were also excavated during the biomass harvests and the evolution of belowground biomass estimated by interpolation. Soil carbon change was estimated from soil samples that were taken each year (except for 2002) at 0-20 cm depth [6,7,8]. For more background on the POP/EUROFACE experiment and its main findings, see a recent review on this topic [9].

As the site was planted on a former agricultural field, the soil was initially very nutrient rich (between 7.7 and 10.4 µg NO_3_^-^ and NH_4_^+^ g^-1^ soil). Tree growth was not nutrient limited during the entire course of the experiment [9]. In this study we therefore only report biomass yields from the unfertilized plot halves. However, these nutrient rich conditions are not likely to persist in the longer term, nor are nutrient rich soils normally used for SRC plantations. To account for the carbon cost of fertilization, in the full life cycle analysis we did include modeled fertilizer production and application costs (Table S1, S2).

**Net energy balance of poplar short rotation coppice (NEB):** We estimated the total energy input costs of a poplar SRC taking into account all processes occurring in the full life cycle of bio-energy production (Table S1, S3, S4 and S5). We estimated the fixed energy costs (i.e. independent of biomass production) as the sum of the one–off energy costs; e.g. the costs of all machinery manufacture, the costs of field preparation (plowing, harrowing), the costs of the planting material and the maintenance costs of the site (mechanical weed removal, irrigation). We also estimated the costs of production and application of insecticides, fungicides and fertilization. We modeled the variable costs of harvesting, chipping and the transportation of the biomass and those of bio-energy production. The estimate of fertilizer usage was based on the amount of nitrogen removed from the site during each harvest. Additional nitrogen input was added to compensate for the loss of nitrogen as N_2_O during fertilization (N_2_O loss was set to 4% of the applied nitrogen fertilization dose) [10].

Harvested and chipped biomass was then converted to electricity and heat in a combined heat and power plant. Energy content of the wood was 19.3 MJ kg^-1^ [11] and did not differ between CO_2_ treatments. The energy efficiency of a combined heat and power plant was assumed to reach 85% and 37% if only electricity is accounted for [12,13]. The fossil fuel counterpart of the biomass CHP was a coal-fired combined heat and power turbine with an efficiency of 85% and a total CO_2_ emission of 121 g CO_2_ MJ^-1^ [13].

**GHG reduction potential of a poplar SRC (GHGB):** we considered the full GHG reduction potential of a poplar SRC to be the sum of the avoided CO_2_ emissions by displacing fossil fuels, the net GHG cost to produce and use the bio-energy, and the CO_2_ equivalent of N_2_O release and CH_4_ oxidation (Table 1). Since coal is one of the most GHG emitting fuels, this reduction potential is probably overestimated, especially for countries with little coal in their energy mix. Therefore, we also calculated the GHGB for replacing a combined heat and power plant fueled by natural gas (Table S2)

**Life cycle analysis under different CO_2_ and management scenarios:** To account for the uncertainties inherent in the estimates of the energy inputs and outputs, we performed a resampling procedure [14] based on a normal distribution of individual costs and conversion factors found in the literature. We assigned an error of 10% to measured biomass production, and 10% to the various estimates of input and output costs (see Table S3, S4 and S5). The full life cycle of a poplar SRC was simulated as a sequence of the following rotation modules: i) first 3 year rotation cycle followed by ii) the second and all subsequent rotation cycles of either 2 or 3 years. In the model run, the initial planting stock was harvested (coppiced) for 6 rotation cycles, independent of the length of the rotation (i.e. the full life cycle varied between 13 and 18 years). We assumed that the measured productivity of the second rotation was repeated during the following rotations.

**Ecosystem model ORCHIDEE-FM:** The global vegetation model, ORCHIDEE (‘ORganizing Carbon and Hydrology In Dynamic Ecosystems’), was used to simulate the terrestrial biogeochemical processes. ORCHIDEE describes the turbulent surface fluxes of CO_2_, water and energy (transpiration, photosynthesis and respiration), and the dynamics of water and carbon pools (soil moisture budget and allocation, growth, mortality, and soil carbon decomposition). Fluxes were calculated each half-hour, and carbon pools were updated each day.

To simulate potential biomass production under current conditions, nutrient and water limitations were switched off in ORCHIDEE-FM. The model was driven by the 2000-2009 climate and an average CO_2_ concentration of 377 ppm until all carbon pools reached a steady-state equilibrium. Potential biomass production under future conditions was similarly obtained by using the 2059-2068 climate and average CO_2_ concentration (577 ppm) data. ORCHIDEE-FM is driven by half-hourly climate data, therefore half-hourly weather and CO_2_ data were generated based on the reanalysis by the National Centre for Environmental Predictions (NCEP), NOAA, USA, monthly climate data sets supplied by the Climatic Research Unit, University of East Anglia, UK, and future climate simulation under the IPCC SRES A1B provided by the Institute Pierre-Simon Laplace (IPSL), France.

In this study ORCHIDEE was extended by a new module that explicitly simulates forest management. Forest representation is detailed from an average tree to an average stand where trees of different diameters compete for a share of total stand growth. A set of rules are defined to calculate the critical tree density, as well as the intensity and frequency of thinning [15]. Three management practices specific to short-rotation coppices have been added to the general thinning-and-clear-cut framework of ORCHIDEE-FM: (1) Coppicing consists in removing all aboveground biomass and letting several stems resprout on each stump. This practice is simulated in ORCHIDEE-FM by an export of all aboveground biomass except for the quantity corresponding to the initial conditions of the next coppicing cycle. This quantity is determined by the standard initial tree circumference distribution of ORCHIDEE-FM. (2) During the final harvest of short-rotation coppices, stumps are uprooted. This practice is simulated in ORCHIDEE-FM by exporting all aboveground and belowground biomass, except for the quantity corresponding to the initial conditions of the next coppicing cycle. (3) Short-rotation coppices may be irrigated. This practice is simulated by guaranteeing 10 mm of rain per day in addition to the actual climatic inputs. As a result, soil moisture is always maximal. The timing of these practices is set to reproduce that of the POP/EUROFACE experiment.

The generic values corresponding to an average temperate broadleaf species in ORCHIDEE-FM were substituted for by physiological parameters specific to poplar: (1) The maximal velocity of carboxylation (Vcmax) and the maximal rate of electron transport (Vjmax) are replaced by the maximum values measured by [16]. (2) The specific leaf area measured by [16] varies with season and position in the canopy. Since ORCHIDEE-FM does not simulate these variations, the standard value is replaced by the average value measured in July. (3) The standard 10,000 stems ha^-1^ of ORCHIDEE-FM is in the range of the number of stems planted at the POP/EUROFACE sites. However, after each coppicing, several stems may resprout from the same stump. Therefore, the initial number of stems is set to 20,000 stems ha^-1^ after the first coppicing, and 40,000 stems ha^-1^ for the remaining coppicing until final harvest. These values correspond to the evolution of number of stems per stump observed by [17].

Three modifications were made to the allocation framework to simulate the highly disturbed conditions of a short-rotation coppice: (1) Pontailler *et al.* [17] report that no resource is allocated to sexual reproduction for several years after coppicing. This lag effect is set to five years in ORCHIDEE-FM, which leads to null allocation to fruits given the short-rotations considered in this study. (2) Allocation to leaves: in ORCHIDEE-FM is greatly constrained by the maximum leaf area index that the stand is allowed to reach any given year (*lai_max_*). The evolution *lai_max_* in ORCHIDEE-FM, a slow growth with stand average height representing the progressive closure of the canopy, does not correspond to field observations for short-rotation coppices. Both [17] and [3] report a strong limitation during the first two years, which is then waved, even after a coppicing event. To simulate this behavior, *lai_max_* increases with the square of stand age for the first two years, reaching the maximum value of the corresponding plant functional type from at age 3. (3) The woody roots grow little after the first coppicing [3]. To simulate this change in allocation, the aboveground to total wood allocation ratio of ORCHIDEE-FM is set to 0.95 after a coppicing event, until the actual aboveground to total wood ratio prescribed by the model is reached.

Following parameterization, aboveground biomass simulations were compared against site-specific observations reported previously [3]. The model results show a reasonable fit against the site data (Fig. S4), supporting our confidence in the regional simulations presented in this study.

**Effects of SRC on soil carbon**

In addition to the field observation, model simulations were used to estimate the soil carbon dynamics following establishment of a SRC on a former forest, grassland and cropland site. According to the standard methodology in global vegetation modelling, a model “spinup” is performed to set the initial conditions of simulations, in particular for soil carbon. For this “spinup”, ORCHIDEE-FM is repeatedly run until all ecosystem carbon and water pools reach a steady-state equilibrium, using the 10 climatic years preceding the establishment of the plantation for climatic input. In order to assess the effect of SRC on soil carbon, three types of “spinups” are used for the POP/EUROFACE site: one with an unmanaged forest, one with a grassland, and one with an irrigated maize field. The SCR itinerary is then applied until a new soil carbon equilibrium is reached, using two sets of climatic conditions: “current climate”, repeating the 2000-2009 climatic years, and “future” climate, repeating the 2059-2068 climatic years predicted by a combination of reanalysis by the National Centre for Environmental Predictions (NCEP), NOAA, USA, monthly climate data sets supplied by the Climatic Research Unit, University of East Anglia, UK, and future climate simulation under the IPCC SRES A1B provided by the Institute Pierre-Simon Laplace (IPSL), France.

BIOME-BGC is a process model describing the carbon, nitrogen and water cycles [18] of land ecosystems. It has been corroborated for a number of hydrological and carbon cycle components as well as for forest management [19,20,21]. The model can be parameterized for seven biomes including evergreen needleleaf, evergreen broadleaf, deciduous needleleaf, deciduous broadleaf forests, shrubs, and grasslands (C3 and C4 type photosynthesis). For this study we used the model version 4.1.1 with the carbon and nitrogen allocation routine from version 4.1. Parameters for deciduous broadleaf forests were optimized from field measurements of net carbon fluxes [22].

To simulate changes in soil carbon associated with conversion of forests and grasslands to poplar plantation we performed spinup simulation of the BIOME-BGC model for deciduous broad leaf forest and C3 grassland. Then transient simulations were performed for a plantation of deciduous broadleaf forest: 1) with temperate climate (mean annual temperature of 8.3 deg C) and atmospheric CO_2_ concentrations of 360 ppm; and 2) with future climate (mean annual temperature of 10.3 deg C) and atmospheric CO_2_ of 720 ppm. Carbon and nitrogen soil pools for transient simulations were initialized using values for forests and grasslands from spinup simulations. We assumed no nitrogen or water limitations on simulated forest growth.

**References**

1. Miglietta F, Peressotti A, Vaccari FP, Zaldei A, De Angelis P, et al. (2001) Free-air CO_2_ enrichment (FACE) of a poplar plantation: the POPFACE fumigation system. New Phytologist 150: 465-476.

2. Calfapietra C, Gielen B, Sabatti M, De Angelis P, Miglietta F, et al. (2003) Do above ground growth dynamics of poplar change with time under CO_2_ enrichment? New Phytologist 160: 305-318.

3. Liberloo M, Calfapietra C, Lukac M, Godbold DL, Luo ZB, et al. (2006) Woody biomass production during the second rotation of a bio-energy *Populus* plantation increases in a future high CO_2_ world. Global Change Biology 12: 1094-1106.

4. Liberloo M, Dillen SY, Calfapietra C, Marinari S, Luo ZB, et al. (2005) Elevated CO_2_ concentration, fertilization and their interaction: growth stimulation in a short-rotation poplar coppice (EUROFACE). Tree Physiology 25: 179-189.

5. Lukac M, Calfapietra C, Godbold DL (2003) Production, turnover and mycorrhizal colonisation of root systems of three *Populus* species grown under elevated CO_2_ (POPFACE). Global Change Biology 9: 838-848.

6. Hoosbeek MR, Li Y, Scarascia-Mugnozza GE (2006) Free atmospheric CO_2_ enrichment (FACE) increased labile and total carbon in the mineral soil of a short rotation Poplar plantation. Plant and Soil 281: 247-254.

7. Hoosbeek MR, Lukac M, van Dam D, Godbold DL (2004) More new carbon in the mineral soil of a poplar plantation under Free Air Carbon Enrichment: cause of increased priming effect? Global Biogeochemical Cycles 18.

8. Hoosbeek MR, Scarascia-Mugnozza GE (2009) Increased litter build up and soil organic matter stabilization in a poplar plantation after 6 years of atmospheric CO_2_ enrichment (FACE): final results of POP-EUROFACE compared to other forest FACE experiments. Ecosystems 12: 220-239.

9. Liberloo M, Lukac M, Calfapietra C, Hoosbeek MR, Gielen B, et al. (2009) Coppicing shifts CO_2_ stimulation of poplar productivity to above-ground pools: a synthesis of leaf to stand level results from the POP/EUROFACE experiment. New Phytologist 182: 331-346.

10. Crutzen PJ, Mosier AR, Smith KA, Winiwarter W (2008) N_2_O release from agro-biofuel production negates global warming reduction by replacing fossil fuels. Atmospheric chemistry and physics 8: 389-395.

11. Luo ZB, Calfapietra C, Liberloo M, Scarascia-Mugnozza G, Polle A (2006) Carbon partitioning to mobile and structural fractions in poplar wood under elevated CO_2_ (EUROFACE) and N-fertilization. Global Change Biology 12: 272-283.

12. Garcia Cidad V, Mathijs E, Nevens F, Reheul D (2003) Energiegewassen in de Vlaamse landbouwsector. 94 p.

13. Sims REH, Hastings A, Schlamadinger B, Taylor G, Smith P (2006) Energy crops: current status and future prospects. Global Change Biology 12: 2054-2076.

14. Rubinstein RY (1981) Simulation and the Monte Carlo Method. New York: John Wiley & Sons. 304 p.

15. Bellasen V, Le Maire G, Dhôte JF, Viovy N, Ciais P Modelling forest management within a global vegetation model - Part 1: model structure and standard results. Ecological Modelling In Review.

16. Calfapietra C, Gielen B, Galema ANJ, Lukac M, De Angelis P, et al. (2003) Free-air CO_2_ enrichment (FACE) enhances biomass production in a short-rotation poplar plantation (POPFACE). Tree Physiology 23: 805-814.

17. Pontailler JY, Ceulemans R, Guittet J (1999) Biomass yield of poplar after five 2-year coppice rotations. Forestry 72: 157-163.

18. Thornton PE (1998) Regional ecosystem simulation: Combining surface- and satellite-based observations to study linkages between terrestrial energy and mass budgets. Missoula: University of Montana.

19. Churkina G, Running SW (2000) Investigating the balance between timber harvest and productivity of the global coniferous forests under global change. Climatic Change 47: 167-191.

20. Churkina G, Tenhunen J, Thornton PE, Elbers JA, Erhard M, et al. (2003) Analyzing the ecosystem carbon dynamics of four european coniferous forests using a biogeochemistry model. Ecosystems 6.

21. Vetter M, Wirth C, Böttcher H, Churkina G, Schulze ED, et al. (2005) Partitioning direct and indirect human-induced effects on carbon sequestration of managed coniferous forests using model simulations and forest inventories. Global Change Biology 11: 810-827.

22. Trusilova K, Trembath J, Churkina G (2009) Parameter estimation and validation of the terrestrial ecosystem model biome-bgc using eddy -covariance flux measurements. Jena. 16 p.

23. Gasol CM, Gabarrell X, Anton A, Rigola M, Carrasco J, et al. (2009) LCA of poplar bioenergy system compared with Brassica carinata energy crop and natural gas in regional scenario. Biomass and Bioenergy 33: 119-129.

24. Heller MC, Keoleian GA, Volk TA (2003) Life cycle assessment of a willow bioenergy cropping system. Biomass and Bioenergy 25: 147-165.

25. De Beer J, Worrell E, Blok K (1998) Future technologies for energy efficient iron and steel making. Annual review of energy and the environment 23: 123-205.

26. Tilman D, Hill J, Lehman C (2006) Carbon negative biofuels from low-input high-diversity grassland biomass. Science 314: 1598-1600.

27. Nielsen V, Sorensen CG (1994) Drift. A programme to calculate the need for labour. Horsens: Statens Jordbrugstekninske forsog. 124 p.

28. Dalgaard T, Halberg N, Porter JR (2001) A model for fossil energy use in Danish agriculture used to compare organic and conventional farming. Agriculture, Ecosystems and Environment 87: 51-65.

29. Lettens S, Muys B, Ceulemans R, Moons E, Garcia J, et al. (2003) Energy budget and greenhouse gas balance evaluation of sustainable coppice systems for electricity production. Biomass and Bioenergy 24: 179-197.

30. Börjesson PII (1996) Energy analysis of biomass production and transportation. Biomass and Bioenergy 11: 305-318.

31. Hülsbergen K-J, Feil B, Biermann S, Rathke G-W, Kalk W-D, et al. (2001) A method of energy balancing in crop production and its application in a long-term fertilizer trial. Agriculture, Ecosystems and Environment 86: 303-321.

32. Frischknecht R (2005) Ecoinvent Data v1.2: from heterogenous database to unified and transparent LCA data. Int J Life Cycle Assesment 10.

33. Sintzoff I, Martin J, Menu JF, Temmerman M, Thiry J, et al. (2001) Woodsustain. Contributions du bois-énergie au développement durable en belgique. Final Summary.

34. Robertson GP, Paul EA, Harwood RR (2000) Greenhouse gases in intensive agriculture: contributions of individual gases to the radiative forcing of the atmosphere. Science 289: 1922-1925.

35. IPCC (2001) Climate change 2001: the scientific basis. Contribution of Working Group I to the Third Assessment Report of the Intergovernmental Panel on Climate Change. Houghton JT, Ding Y, Griggs DJ, Noguer M, van der Linden PJ et al., editors. Cambridge: Cambridge University Press. 881 p.

36. Hill J, Nelson E, Tilman D, Polasky S, Tiffany D (2006) Environmental, economic, and energetic costs and benefits of biodiesel and ethanol biofuels. PNAS 103: 11206-11210.

37. Schmer MR, Vogel KP, Mitchell RB, Perrin RK (2008) Net energy of cellulosic ethanol from switchgrass. PNAS 105: 464-469.
