# Supplementary material for: Bio-Energy Retains Its Mitigation Potential Under Elevated CO2
Source: PLoS One. 2010 Jul 19;5(7):e11648. doi: 10.1371/journal.pone.0011648 (PMC2906505; doi:10.1371/journal.pone.0011648)
Supplement: Table S1 — Components of the full life cycle analysis of poplar SRC for biomass production under current ambient and future elevated [CO2] and subsequent combustion in a combined heat and power plant. Avoided CO2 emissions, GHGB and mitigation potential were based on the assumption that coal was substituted by biomass in a combined heat and power plant. Excluding soil carbon dynamics (see Table 2). (0.12 MB DOC) [file pone.0011648.s002.doc]

**Table S1:** Components of the full life cycle analysis of poplar SRC for biomass production under current ambient and future elevated [CO_2_] and subsequent combustion in a combined heat and power plant. Avoided CO_2_ emissions, GHGB and mitigation potential were based on the assumption that **coal** was substituted by biomass in a combined heat and power plant. Excluding soil carbon dynamics (see Table 2)

|  | Current ambient CO_2_, 18 years life cycle | | Future Elevated CO_2_, 18 years life cycle | | Current Ambient CO_2_, 13 years life cycle | | Future Elevated CO_2_, 13 year life cycle | |
| --- | --- | --- | --- | --- | --- | --- | --- | --- |
|  | Mean | Std | Mean | Std | Mean | Std | Mean | Std |
| **Biological production** |  |  |  |  |  |  |  |  |
| Biomass production (t DM ha^-1^) | 415 | 17 | 491 | 20 | 322 | 13 | 451 | 19 |
| Mean annual biomass prod (t DM ha^-1^ yr^-1^) | 23 | 1 | 27 | 1 | 27 | 1 | 38 | 2 |
| **Energy production** |  |  |  |  |  |  |  |  |
| Gross energy in biomass (GJ ha^-1^) | 8002 | 329 | 9481 | 393 | 6209 | 254 | 8709 | 359 |
| Gross energy CHP (GJ ha^-1^) | 6801 | 280 | 8058 | 334 | 5278 | 216 | 7403 | 305 |
| Gross energy from CHP electricity (GJ ha^-1^) | 2801 | 115 | 3318 | 138 | 2173 | 89 | 3048 | 126 |
| Net energy balance (GJ ha^-1^) | 7432 | 308 | 8784 | 367 | 5757 | 238 | 8066 | 335 |
| Net energy balance CHP (GJ ha^-1^) | 6232 | 258 | 7362 | 308 | 4825 | 200 | 6759 | 281 |
| Net energy balance CHP electricity (GJ ha^-1^) | 2231 | 94 | 2622 | 112 | 1721 | 73 | 2405 | 102 |
| **Energy consumption** |  |  |  |  |  |  |  |  |
| Water Use (GJ ha^-1^) | 288 | 14 | 372 | 18 | 224 | 11 | 341 | 17 |
| Harvest and chipping (GJ ha^-1^) | 120 | 7 | 142 | 8 | 93 | 5 | 131 | 8 |
| Transport (GJ ha^-1^) | 70 | 4 | 84 | 5 | 55 | 3 | 77 | 4 |
| Fertilizer production (GJ ha^-1^) | 42 | 3 | 50 | 4 | 33 | 2 | 46 | 3 |
| Production cost of machinery (GJ ha^-1^) | 37 | 7 | 37 | 8 | 37 | 8 | 37 | 7 |
| Application insecticide fungicide (GJ ha^-1^) | 0.8 | 0.3 | 0.8 | 0.3 | 0.8 | 0.3 | 0.8 | 0.3 |
| Stump kill and removal (GJ ha^-1^) | 0.3 | 0.2 | 0.3 | 0.2 | 0.3 | 0.2 | 0.3 | 0.2 |
| Mechanical weed control (GJ ha^-1^) | 0.3 | 0.1 | 0.3 | 0.1 | 0.3 | 0.1 | 0.3 | 0.1 |
| Production insecticide (GJ ha^-1^) | 1.2 | 0.6 | 1.1 | 0.6 | 1.1 | 0.6 | 1.1 | 0.6 |
| Ploughing (GJ ha^-1^) | 0.49 | 0.04 | 0.49 | 0.04 | 0.49 | 0.04 | 0.49 | 0.04 |
| Production fungicide (GJ ha^-1^) | 1.01 | 0.26 | 1.01 | 0.26 | 1.01 | 0.26 | 1.01 | 0.26 |
| Application fertiliser (GJ ha^-1^) | 0.74 | 0.17 | 0.74 | 0.17 | 0.74 | 0.17 | 0.74 | 0.17 |
| Cuttings production (GJ ha^-1^) | 2.94 | 0.24 | 2.94 | 0.24 | 2.94 | 0.24 | 2.94 | 0.24 |
| Planting (GJ ha^-1^) | 0.74 | 0.06 | 0.74 | 0.06 | 0.74 | 0.06 | 0.74 | 0.06 |
| Harrowing (GJ ha^-1^) | 2.35 | 0.47 | 2.35 | 0.46 | 2.35 | 0.46 | 2.35 | 0.46 |
| Total cost (GJ ha^-1^) | 570 | 25 | 696 | 30 | 453 | 20 | 643 | 28 |
| **Consumption production ratio** |  |  |  |  |  |  |  |  |
| Energy ratio biomass | 14 | 1 | 14 | 1 | 14 | 1 | 14 | 1 |
| Energy ratio CHP | 11 | 1 | 11 | 1 | 11 | 1 | 11 | 1 |
| Energy ratio CHP electricity | 4 | 1 | 4 | 1 | 4 | 1 | 4 | 1 |
| **Avoided emissions** |  |  |  |  |  |  |  |  |
| Avoided CO_2_ emission CHP (ton CO_2_ ha^-1^) | 701 | 29 | 830 | 34 | 544 | 22 | 762 | 31 |
| Electricity from CHP (ton CO_2_ ha^-1^) | 339 | 14 | 401 | 17 | 263 | 11 | 369 | 15 |
| CH4 oxidation (ton CO_2_ equi ha^-1^) | 0.05 | 0.00 | 0.05 | 0.00 | 0.05 | 0.00 | 0.05 | 0.00 |
| **CO_2_ emissions** |  |  |  |  |  |  |  |  |
| Electricity use (ton CO_2_ ha^-1^) | 41 | 2 | 53 | 3 | 32 | 2 | 48 | 3 |
| Diesel use (ton CO_2_ ha^-1^) | 19 | 1 | 21 | 1 | 15 | 1 | 20 | 1 |
| N_2_O Emissions (ton CO_2_ equi ha^-1^) | 14 | 1 | 17 | 1 | 11 | 1 | 16 | 1 |
| Mixed sources (ton CO_2_ ha^-1^) | 2.0 | 0.1 | 2.3 | 0.2 | 1.5 | 0.1 | 2.2 | 0.2 |
| **GHG balance** |  |  |  |  |  |  |  |  |
| CHP (ton CO_2_ equi ha^-1^) | 35 | 1 | 41 | 2 | 37 | 2 | 52 | 2 |
| Electricity from CHP (ton CO_2_ equi ha^-1^) | 15 | 1 | 17 | 1 | 16 | 1 | 22 | 1 |
| **Mitigation potential** |  |  |  |  |  |  |  |  |
| CHP (ton CO_2_ equi MJ^-1^) | 84 | 1 | 84 | 1 | 84 | 1 | 84 | 1 |
| Electricity from CHP (ton CO_2_ equi MJ^-1^) | 35 | 1 | 35 | 1 | 35 | 1 | 35 | 1 |
