# Supplementary material for: Bio-Energy Retains Its Mitigation Potential Under Elevated CO2
Source: PLoS One. 2010 Jul 19;5(7):e11648. doi: 10.1371/journal.pone.0011648 (PMC2906505; doi:10.1371/journal.pone.0011648)
Supplement: Table S2 — Components of the life cycle analysis of poplar SRC for biomass production under current ambient and future elevated [CO2] and subsequent combustion in a combined heat and power plant. Avoided CO2 emissions, GHGB and mitigation potential were based on the assumption that gas was substituted by biomass in the combined heat and power plant. Excluding soil carbon dynamics (see Table 2). (0.05 MB DOC) [file pone.0011648.s003.doc]

**Table S2** Components of the life cycle analysis of poplar SRC for biomass production under current ambient and future elevated [CO_2_] and subsequent combustion in a combined heat and power plant. Avoided CO_2_ emissions, GHGB and mitigation potential were based on the assumption that **gas** was substituted by biomass in the combined heat and power plant. Excluding soil carbon dynamics (see Table 2)

|  | Current ambient CO_2_, 18 years life cycle | | Future Elevated CO_2_, 18 years life cycle | | Current Ambient CO_2_, 13 years life cycle | | Future Elevated CO_2_, 13 years life cycle | |
| --- | --- | --- | --- | --- | --- | --- | --- | --- |
|  | Mean | Std | Mean | Std | Mean | Std | Mean | Std |
| **Avoided CO_2_ emissions** |  |  |  |  |  |  |  |  |
| CHP (ton CO_2_ ha^-1^) | 401 | 17 | 475 | 20 | 311 | 13 | 437 | 18 |
| Electricity from CHP (ton CO_2_ ha^-1^) | 196 | 8 | 232 | 10 | 152 | 6 | 213 | 9 |
| CH4 oxidation (ton CO_2_ equi ha^-1^) | 0.05 | 0.01 | 0.05 | 0.01 | 0.05 | 0.01 | 0.05 | 0.01 |
| **CO_2_ emissions** |  |  |  |  |  |  |  |  |
| Electricity use (ton CO_2_ ha^-1^) | 41 | 2 | 53 | 3 | 32 | 2 | 48 | 3 |
| Diesel use (ton CO_2_ ha^-1^) | 19 | 1 | 21 | 1 | 15 | 1 | 20 | 1 |
| N_2_O Emissions (ton CO_2_ equi ha^-1^) | 14 | 1 | 17 | 1 | 11 | 1 | 16 | 1 |
| Mixed sources (ton CO_2_ ha^-1^) | 2.0 | 0.1 | 2.3 | 0.2 | 1.5 | 0.1 | 2.2 | 0.2 |
| **GHG balance** |  |  |  |  |  |  |  |  |
| CHP (ton CO_2_ equi ha^-1^) | 18 | 1 | 21 | 2 | 19 | 1 | 27 | 1 |
| Electricity from CHP (ton CO_2_ equi ha^-1^) | 7 | 1 | 8 | 1 | 7 | 1 | 10 | 1 |
| **Mitigation potential** |  |  |  |  |  |  |  |  |
| CHP (ton CO_2_ equi MJ^-1^) | 44 | 1 | 43 | 1 | 44 | 1 | 43 | 1 |
| Electricity from CHP (ton CO_2_ equi MJ^-1^) | 16 | 1 | 16 | 1 | 16 | 1 | 16 | 1 |
