# Supplementary material for: Bio-Energy Retains Its Mitigation Potential Under Elevated CO2
Source: PLoS One. 2010 Jul 19;5(7):e11648. doi: 10.1371/journal.pone.0011648 (PMC2906505; doi:10.1371/journal.pone.0011648)
Supplement: Table S3 — Estimated fixed energy costs (GJ ha-1) for planting, growing and maintaining a poplar SRC. (0.05 MB DOC) [file pone.0011648.s004.doc]

**Table S3:** Estimated fixed energy costs (GJ ha^-1^) for planting, growing and maintaining a poplar SRC.

| Fixed energy costs (GJ ha^-1^ application^-1^) | | number of applications | | Comment |
| --- | --- | --- | --- | --- |
|  |  | 18 years | 13 years |  |
| Production cost of machinery^*^ | 37.5 | 1 | 1 | We estimated the weight of the machinery used in field preparation, planting and maintaining the plantation (139 hp tractor, 50 hp tractor, implements used for plowing, harrowing, planting, harvesting, weed removal, insecticide and fungicide treatment and fertilization) to be 16 tons [23,24]. We assumed all machinery was made of steel and estimated the steel manufacture cost at 37.5 MJ kg^-1^ [25,26]. We divided the production cost of the machinery across an average European farm size of 16 ha (European Commission: agricultural development http://ec.europa.eu/agriculture/faq/index_nl.htm), and assumed the machinery has an average lifetime equal to the lifetime of the plantation. |
| Soil preparation |  |  |  | Soil preparation included plowing and harrowing before winter and planting cuttings in early spring. Mechanical weed removal was applied at the start of each rotation cycle and estimated to consume 2 liters of diesel fuel ha^-1^ [27]. We assumed that 20.0 and 6.3 l ha^-1^ of diesel fuel were used for plowing and harrowing [28]. Diesel has a total energy cost (including production cost) of 40.9 GJ l^-1^ [12]. We assumed it costs 1.14 GJ ha^-1^ to plant the poplar cuttings [23,29] |
| Plowing | 0.82 | 1 | 1 |
| Harrowing | 0.26 | 1 | 1 |
| Planting | 1.14 | 1 | 1 |
| Mechanical weed control | 0.08 | 6 | 6 |
| Cuttings production | 0.3 | 1 | 1 | We assumed it costs 0.3 GJ ha^-1^ [30] to produce the cuttings. |
| Production |  |  |  | We assumed that 0.5 kg ha^-1^ of insecticide and fungicide [23] is applied during each rotation cycle, which on average cost 0.292 GJ kg^-1^ and 0.218 GJ kg^-1^ to produce, respectively [28,31]. |
| insecticide | 0.15 | 6 | 6 |
| fungicide | 0.11 | 6 | 6 |
| Application |  |  |  | We assumed it costs 6 l diesel ha^-1^ to spray fungicide or insecticide on the poplar plantation [23], and 3 l diesel ha^-1^ to spread top-fertiliser on the soil [23] |
| Insecticide fungicide | 0.245 | 12 | 12 |
| fertiliser | 0.123 | 6 | 6 |
| Stump kill down and removal | 2.35 | 1 | 1 | Stumps and coarse root systems are destroyed and removed at the end of the life cycle of a poplar SRC at an estimated cost of 57 l diesel ha^-1^ [23]. |
