# Supplementary material for: Bio-Energy Retains Its Mitigation Potential Under Elevated CO2
Source: PLoS One. 2010 Jul 19;5(7):e11648. doi: 10.1371/journal.pone.0011648 (PMC2906505; doi:10.1371/journal.pone.0011648)
Supplement: Table S4 — Machinery utilized for field operations in a poplar SRC. Data from [24] and [23]. (0.03 MB DOC) [file pone.0011648.s005.doc]

**Table S4:** Machinery utilized for field operations in a poplar SRC. Data from [24] and [23].

| Activity | Machines | Weight (kg) | Power (kW) | hp |
| --- | --- | --- | --- | --- |
|  | **tractor** | 6300 | 104 | 139 |
| Ploughing | implement | 1226 |  |  |
| Harrowing | implement | 1053 |  |  |
| Planting | implement | 1400 |  |  |
| Harvesting (Maskiner bender) | implement | 1250 |  |  |
|  | **tractor** | 2572 | 37 | 50 |
| Mechanical weed removing | implement | 500 |  |  |
| Insecticide/fungicide treatment | implement (boom sprayer) | 670 |  |  |
| Fertilization treatment | implement (spreader) | 1000 |  |  |
| Total weight machinery |  | 15971 |  |  |
