# Supplementary material for: Bio-Energy Retains Its Mitigation Potential Under Elevated CO2
Source: PLoS One. 2010 Jul 19;5(7):e11648. doi: 10.1371/journal.pone.0011648 (PMC2906505; doi:10.1371/journal.pone.0011648)
Supplement: Table S5 — Estimated variable energy costs. (0.04 MB DOC) [file pone.0011648.s006.doc]

**Table S5:** Estimated variable energy costs.

| Variable costs | | Units | Comments |
| --- | --- | --- | --- |
| Irrigation:  -current ambient  -future elevated | 0.634  0.793 | GJ ton^-1^ DM ha^-1^ yr^-1^ | Irrigation of the experimental plots at the POP/EUROFACE site consumed on average 15.75 GJ ha^-1^ yr^-1^. Energy was provided by low voltage electricity mix for the EU which emits 0.142 kg CO2 MJ^-1^ electricity [32]. |
| Fertilizer production | 49.28 | GJ ton^-1^ N, P, K fertilizer | We assumed the plantation was fertilized during each rotation cycle, according to the amount of N removed from the site during the previous harvest (i.e. between 140 and 280 kg N-P-K fertilizer 20-6-6 per ha). We estimated the average cost to produce fertilizer at 49.28 GJ ton^-1^ N from[31], and [28]. Poplar wood contained on average 0.2% N, and the N content was not affected by the CO_2_ treatment[11]. Further, we assumed the use of ammonium sulphate as N fertilizer. The CO2 emission from its production is 2.3 kg CO2 kg N^-1^. Other N fertilizers produce between 1.15 and 5.73 kg CO2 kg N^-1^ [32].. |
| Harvesting and chipping | 0.29 | GJ ton DM^-1^ | From [33]. The chips are stored on average for 180 days. Storage is in the open air and thus requires no energy input. |
| Transport | 0.0085 | GJ ton DM^-1^ km^-1^ | We assume woody biomass was transported from the site to the power plant for conversion into usable energy over a distance of 20 km. Conversion factor from [30]. |
| Efficiency of CHP | 0.85 | Dimensionless | The efficiency to produce electricity is 0.35 the total efficiency including heat production is 0.85 [13]. |
| Emission from diesel | 0.078 | kg CO_2_ GJ^-1^ | The energetic costs (GJ) of harvest, chipping, transport and all fixed costs were converted into CO_2_ emissions, using the full life cycle CO_2_ cost of diesel from [13] |
| N_2_O emmissions |  | ton CO_2_-equivalent ha^-1^ | N_2_O emission from fertilization was calculated from the amount of fertilizer added (see Table S3) and a N_2_O output/fixed N input ratio of 4% [10]. A global warming potential on a 20-year time horizon was used. |
| CH_4_ |  | ton CO_2_-equivalent ha^-1^ | Values for CH_4_ mitigation were taken from [34]: CH_4_ oxidation attributes a global warming mitigation potential of 5 g CO_2_ equivalent m^-2^ yr^-1^, using IPCC conversion factors on a 20 year time horizon [35]. |
