# Supplementary material for: Bio-Energy Retains Its Mitigation Potential Under Elevated CO2
Source: PLoS One. 2010 Jul 19;5(7):e11648. doi: 10.1371/journal.pone.0011648 (PMC2906505; doi:10.1371/journal.pone.0011648)
Supplement: Table S6 — Greenhouse gas mitigation potential (i.e. GHG sequestration (positive) or release (negative) per net energy gain (g CO2 equivalent MJ-1) for different bio-fuels and their respective fossil fuel counterparts. The mitigation potential of a biofuel production system can be quantified by the ratio of its GHGB and NEB, and is thus a measure of the amount of avoided greenhouse gas emissions (CO2-equivalent) for every net MJ of energy produced. (0.04 MB DOC) [file pone.0011648.s007.doc]

**Table S6:** Greenhouse gas mitigation potential (i.e. GHG sequestration (positive) or release (negative) per net energy gain (g CO_2_ equivalent/MJ) for different bio-fuels and their respective fossil fuel counterparts. The mitigation potential of a biofuel production system can be quantified by the ratio of its GHGB and NEB, and is thus a measure of the amount of avoided greenhouse gas emissions (CO_2_-equivalent) for every net MJ of energy produced.

| Bio-energy system | GHG mitigation potential (g CO_2_ equi./MJ) | Reference system | GHG mitigation potential (g CO_2_ equi./MJ) | Source |
| --- | --- | --- | --- | --- |
| Corn grain ethanol | -85 | Gasoline | -97 | [36] |
| Soybean biodiesel | -82 | Diesel | -49 | [36] |
| low input high diversity grasslands (LIHD) electricity | 557 | Coal generated electricity | -290 | [26] |
| LIHD ethanol | 346 | Gasoline | -97 | [26] |
| LIHD synfuel | 339 | 38% gasoline + 62% diesel | -82 | [26] |
| Switchgrass ethanol | -6 | Gasoline | -94 | [37] |
| Poplar SRC combined heat and power (CHP) | 84 | Coal CHP | -103 | This study & [13] |
| Poplar SRC CHP electricity only | 35 | Coal CHP electricity only | -121 | This study & [13] |
| Poplar SRC CHP | 44 | Gas CHP | -59 | This study & [13] |
| Poplar SRC CHP electricity only | 16 | Gas CHP electricity only | -70 | This study & [13] |
