# Supplementary figures and images for: Bio-Energy Retains Its Mitigation Potential Under Elevated CO2
Source: PLoS One. 2010 Jul 19;5(7):e11648. doi: 10.1371/journal.pone.0011648 (PMC2906505; doi:10.1371/journal.pone.0011648)

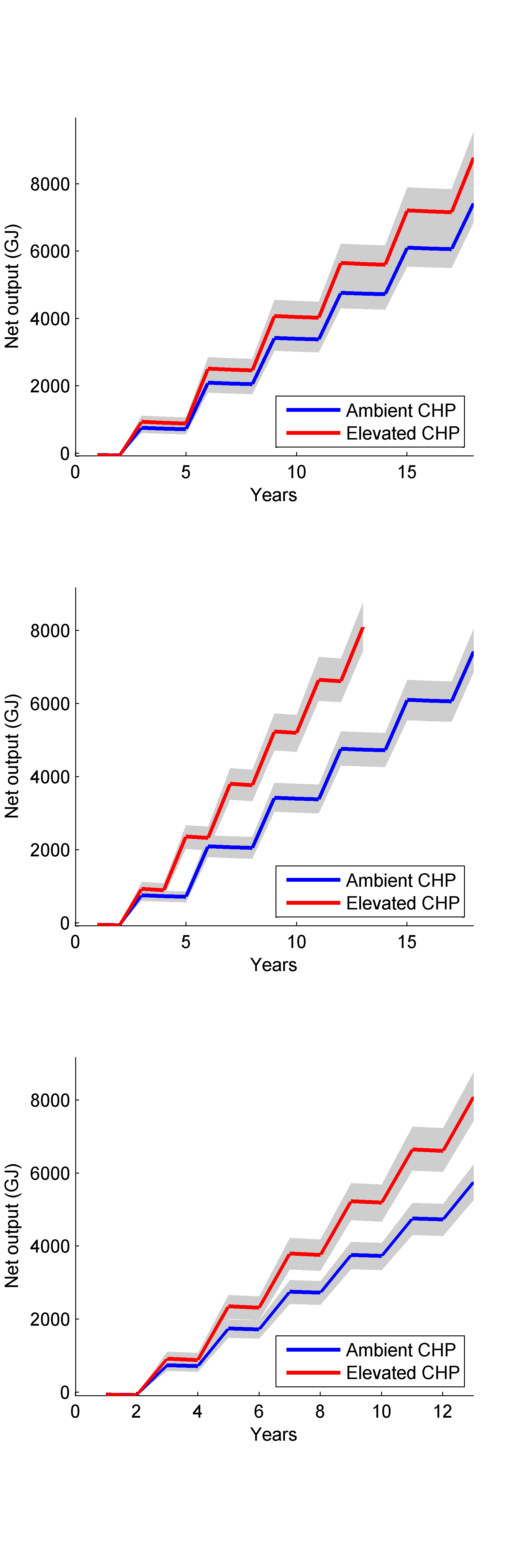

Supplement: Figure S1 — Simulated life cycle of net energy gain (GJ) from a poplar SRC growing for 6 rotations under different coppice regimes in current and elevated [CO2]. a) Current and elevated [CO2] grown poplars managed in three year rotations b) current [CO2] grown poplars managed in three year rotation cycles, elevated [CO2] grown trees in two year rotation cycles, c) both current and elevated [CO2] grown trees are managed in two year rotation cycles. The grey area shows the 95% uncertainty interval. (0.95 MB TIF) [file pone.0011648.s008.tif]

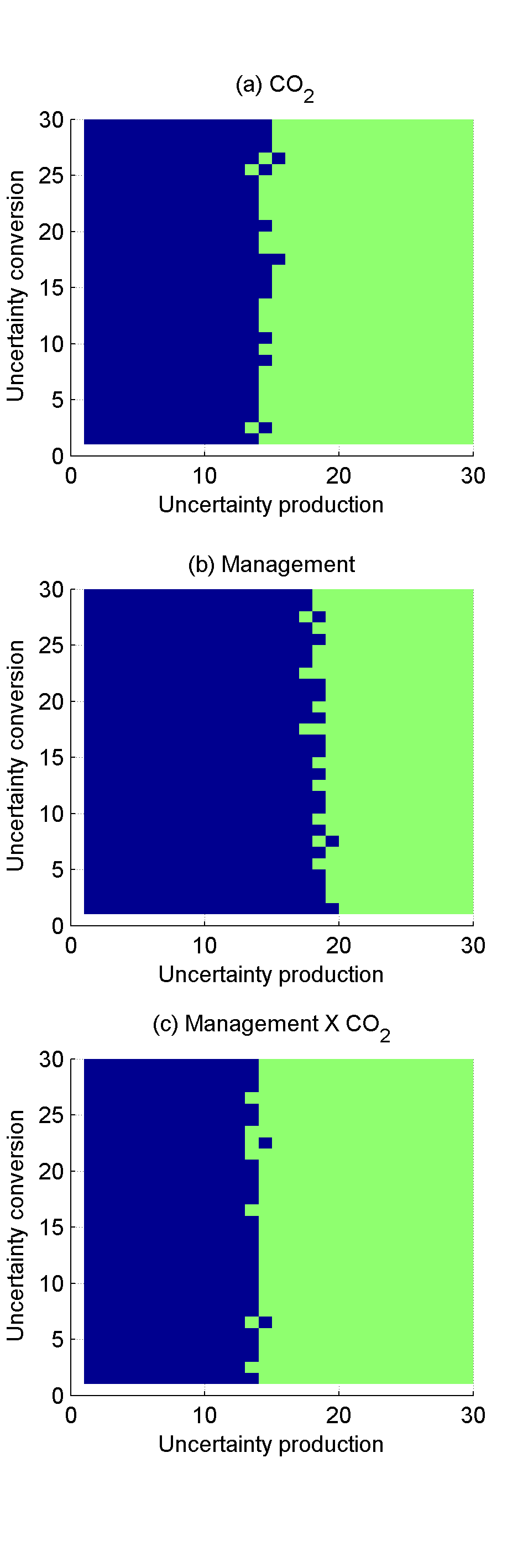

Supplement: Figure S2 — Sensitivity analysis of NEB to uncertainties in biomass production and conversion factors (Tables S1, S3, S4, S5). (a) Blue pixels shows uncertainty settings for which NEB under elevated is significantly higher than NEB under current [CO2]. Green pixels show uncertainty settings for which no significant differences were found. (b) Similarly, blue pixels show uncertainty settings for which NEB for two year rotations is significantly higher than NEB for three year rotations. (c) Whether [CO2] and management have a significant effect on the NEB of an SRC depends on the uncertainty in biomass production. Uncertainties in biomass production below 15% will result in a significant effect, irrespective of the uncertainty in the conversion. (3.87 MB TIF) [file pone.0011648.s009.tif]

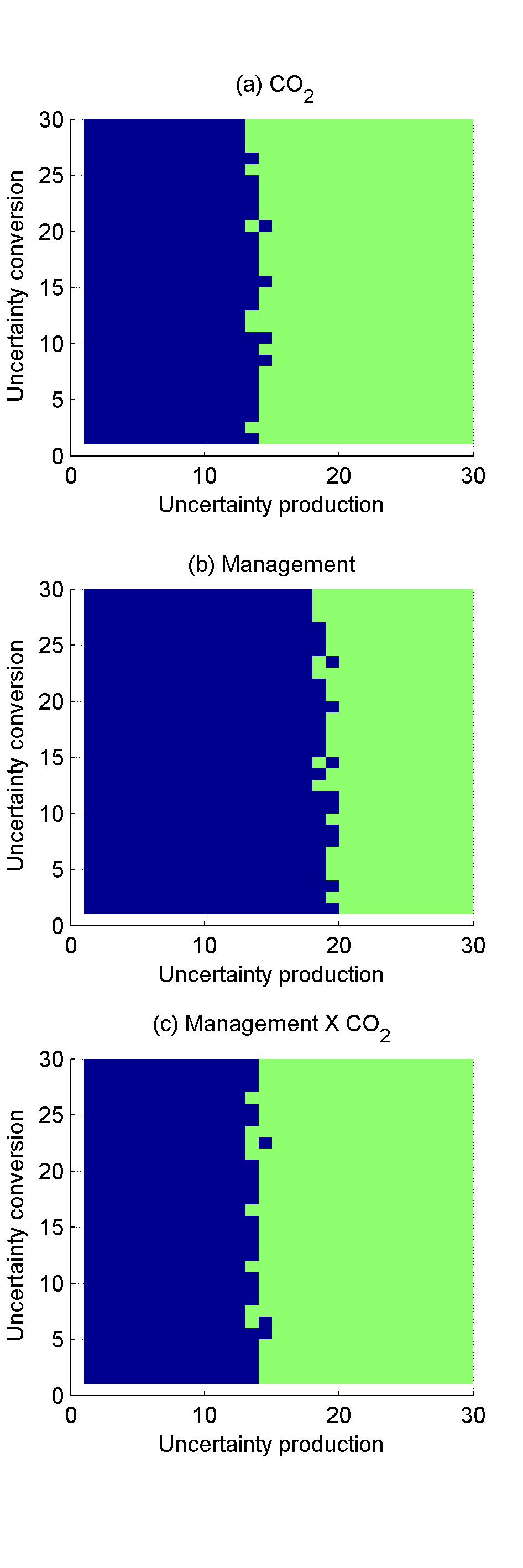

Supplement: Figure S3 — Sensitivity analysis of the GHGB to uncertainties in biomass production and conversion factors (Tables S1, S3, S4 and S5). In the combined heat and power plant coal is substituted by biomass. (a) Blue pixels show uncertainty settings for which poplar SRC under elevated [CO2] removes significantly more CO2-equivalents from the atmosphere than under current [CO2]. Green pixels shows uncertainty settings for which no significant differences were found. (b): Settings for which GHGB for two year rotations is significantly higher than GHGB for three year rotations (c) Blue pixels show uncertainty settings for which poplar SRC under elevated [CO2] and with two year rotations removes significantly more CO2-equivalent from the atmosphere than under current [CO2] with three year rotations. (3.87 MB TIF) [file pone.0011648.s010.tif]

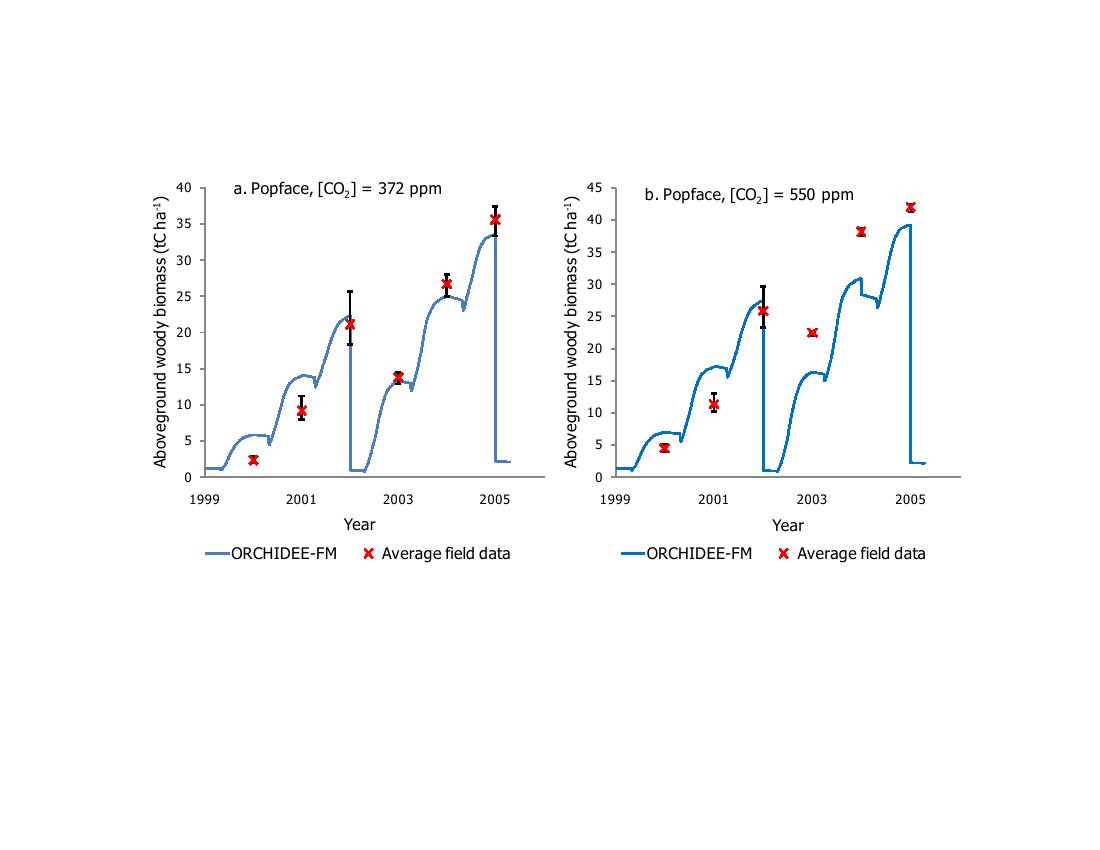

Supplement: Figure S4 — Comparison of model output of ORCHIDEE-FM against observed aboveground biomass production. (a) Comparison for ambient [CO2] (b) Comparison for elevated [CO2]. Small decreases in biomass are due to modeled reserve mobilization to subsidize growth in the following spring. The larger decrease in biomass in the second year of the second rotation is due to the onset of competition, the current model version, accounts for this loss of biomass on the last day of the year. For both comparisons, the climate data driving simulations come from the 0.25° resolution REMO reanalysis, which covers Europe from 1861 to 2007. (0.10 MB TIF) [file pone.0011648.s011.tif]
